# Auditory local–global temporal processing: evidence for perceptual reorganization with musical expertise

**DOI:** 10.1038/s41598-020-72423-7

**Published:** 2020-10-02

**Authors:** Patrick Susini, Sarah Jibodh Jiaouan, Elena Brunet, Olivier Houix, Emmanuel Ponsot

**Affiliations:** 1 STMS Ircam-CNRS-SU, 1 Place Igor Stravinsky, 75004 Paris, France; 2 Laboratoire des systèmes perceptifs, Département d’études cognitives, École normale supérieure, PSL University, CNRS, 29 rue d’Ulm, 75005 Paris, France

**Keywords:** Human behaviour, Perception, Auditory system, Sensory processing, Learning and memory

## Abstract

The way the visual system processes different scales of spatial information has been widely studied, highlighting the dominant role of global over local processing. Recent studies addressing how the auditory system deals with local–global temporal information suggest a comparable processing scheme, but little is known about how this organization is modulated by long-term musical training, in particular regarding musical sequences. Here, we investigate how non-musicians and expert musicians detect local and global pitch changes in short hierarchical tone sequences structured across temporally-segregated triplets made of musical intervals (local scale) forming a melodic contour (global scale) varying either in one direction (monotonic) or both (non-monotonic). Our data reveal a clearly distinct organization between both groups. Non-musicians show global advantage (enhanced performance to detect global over local modifications) and global-to-local interference effects (interference of global over local processing) only for monotonic sequences, while musicians exhibit the reversed pattern for non-monotonic sequences. These results suggest that the local–global processing scheme depends on the complexity of the melodic contour, and that long-term musical training induces a prominent perceptual reorganization that reshapes its initial global dominance to favour local information processing. This latter result supports the theory of “analytic” processing acquisition in musicians.

## Introduction

The way the visual system processes global vs. local scales of spatial information has been addressed for a long time^1^, first under the metaphorical terms: *Which dominates visual perception, the forest or the trees?* Controlled laboratory experiments were conducted to characterize the local–global processing organization of the visual system, employing hierarchical visual patterns arranged spatially and presented simultaneously, such as large characters (“global” scale) made out of smaller characters (“local” scale), either congruent (same characters) or incongruent (different characters); for a review, see^2^. Results from these studies exhibited two main patterns. First, observers are faster and more accurate to identify global rather than local information, an effect referred to as “global advantage”. Second, in incongruent configurations, the global form disrupts the identification of the local form, but the local form has no measurable impact on the processing of the global form; an effect referred to as “global-to-local interference”. The combination of these two effects is often referred to as “the global precedence effect”: the global processing of a hierarchical visual stimulus both *precedes* and strongly *interacts* with its local analysis. The global precedence effect is observed under many different experimental conditions (stimuli, tasks), and is considered by many as a modern support to the Gestaltists’ claim about the primacy of holistic processing^2^. However, some studies have also shown negative results or opposite patterns, revealing that there are circumstances under which a local precedence effect can arise, e.g. depending on how stimuli are constructed^2–4^ or depending on the population tested. For instance, local advantage and local-to-global interference effects have been observed^5^ in individuals with autism spectrum disorders (ASDs). Thus, while the global precedence effect seems to reflect an important property of how visual processing is initially structured, this organization remains plastic, and global processing can in some conditions also be affected by incongruent local information.

In comparison, fewer studies were conducted in the auditory domain to explore the organizational principles of local–global processing. While studies in vision primarily considered the hierarchical processing of spatial elements across space, studies in audition examined the organization of pitch processing across time, arguing that pitch and time are for audition what shape and space are for vision^6,7^.

By investigating the left/right cortical hemispheric specialization for analytic/holistic processing of melodic information in musicians vs. non-musicians, Bever and Chiarello^8^ addressed the first questions related to local–global organization in the auditory domain. Later, Peretz^9^ assessed the ability of listeners to detect changes in melodies occurring either on a single musical interval (“local” scale) or on the whole melodic contour (“global” scale). Comparing brain-damaged populations with either left or right hemisphere damage (LHD or RHD), this study observed that, in LHDs, access to the local information but not to the global information was impaired, whereas in RHDs, access to both global and local information were impaired. These results revealed an hemispheric specialization of local and global levels of processing, with local processing (interval) depending on the capacities to process global information (contour), suggesting for the first time the presence of a global-to-local interference effect in the auditory domain.

Based on the observation that local and global levels of the musical stimuli used in Peretz’ study^9^ could not be manipulated independently (because a contour modification inherently involved an interval change; see^10^), Justus and List^6^ designed a new set of stimuli allowing pitch manipulations either at a local level (on one note in a short temporal window) and/or a global level (on a group of notes in a longer temporal window) in an *independent* manner. These stimuli consist of simple hierarchically structured 9-note sequences, allowing a more direct comparison of local–global organization results obtained from the visual stimuli of Navon’s study^1^. Most studies that followed employed these controlled local–global hierarchical stimuli, in which listeners’ task was to determine whether the local (or global) information was going up/down in pitch^10–12^.

To our knowledge, Bouvet et al.^11^ is the first study that specifically examined the “global precedence effect” in the auditory domain from a psychophysical perspective, and tested the same participants in local–global visual (following^1^) and auditory tasks (following^6^). Results revealed that participants were both faster and more accurate for processing global (letter or pitch) information, with clear global-to-local interference effects. Most importantly, subject-by-subject global-to-local interference effects were correlated across the visual and the auditory tasks, suggesting that local–global processing in vision and audition might be mediated by the same general organizational principles. Yet, music learning constitutes an extensive training that is unique to the auditory modality and yields major enhancement in processing skills e.g. to encode short melodies or to detect deviant tones (e.g. ^13,14^). One can thus ask whether the auditory global precedence effect is modulated by perceptual expertise. Along these lines, Ouimet et al.^12^ examined the effects of extensive musical experience on local–global auditory processing organization by comparing non-musician and musician participants in the same task. Beyond the fact that musicians outperformed non-musicians, their results interestingly showed that musicians exhibited a reduced global advantage compared to non-musicians due to an enhanced ability to process local information, consistent with performance measures from other studies employing interval-contour melodies^15^. Black et al.^10^ replicated this result, and further showed that the global-to-local interference effect on accuracy was reduced for participants with high musical expertise. These results suggest that the local–global auditory processing organization is modulated by musical expertise: expert listeners develop a better ability to process local information and to overcome the initial global interference.

However, several methodological choices for the design of stimuli and experimental procedure might have provided an underestimated view of the actual local–global processing reorganization in musicians. First, melodic sequences used in these studies were constructed using non-integers pitch intervals of 147 cents, in the purpose of reducing “any advantage that might come from musicians’ familiarity with Western intervals or having perfect pitch”^12^. Although interesting to assess how musical training impacts and transfer to general auditory abilities, this experimental choice might not be optimal to maximally expose the reorganization acquired to process musical sequences following specific pitch intervals (i.e. with differences of 50 or 100 cents, corresponding to semi-tones and tones, respectively). Second, while the 9-note sequences used were theoretically organized with local elements (triplets), all notes were equally spaced along the time axis. The absence of timing cues to extract the different local triplets might have prevented musicians to fully benefit from their training to extract and memorize local interval variations^16,17^. Thus, it remains unknown whether the global advantage and global-to-local interference effects are at all observable for musicians presented with local–global melodies organized in temporally-segregated triplets, where local and global information can be parsed more easily. Third, conclusions from previous studies were mainly derived from analyses based on either response times or participants’ performance scores. Performance scores’ analyses, in particular, do not allow distinguishing whether effects are driven by differences in sensitivity of the perceptual system to detect changes at local vs. global levels, or by specific response strategies deployed by listeners, as can more usefully scrutinized using Signal Detection Theory (SDT)^18^. This dissociation is however critical, specifically in an attempt to compare musicians and non-musicians, which may either differ by their processing efficiency and/or by the acquisition of novel judgment strategies. Last, a fourth limitation concerns the intrinsic difference between visual and auditory stimuli: while visual elements are presented simultaneously, by their nature pitch sequences necessarily unfold over time. Black et al.^10^ already pointed out that, with such auditory stimuli, “participants received relevant decision-making information at different times on global and local trials (i.e., global trials at the fourth note and local trials at the second note)” (p. 14). Although latest studies redefined reaction times for these two conditions with respect to the moment when information becomes available (2nd note for the local information, 4th note for the global information) in an attempt to address this issue, they did not control for listeners strategies; some listeners may listen to the whole sequence, before deciding whether a local or a global change was presented, whereas other listeners may decide as soon as the 4th note is heard^19^. Such strategies might have biased any musicians vs. non-musicians comparison. As such, by using stimuli in which the local changes could be accessed before the global information was available, previous studies might have underestimated the extent to which local and global processes interact.

The main purpose of the present study was to re-address the question of local–global reorganization induced by extensive musical training while overcoming the previously evidenced methodological issues. We used a novel experimental paradigm based on a two-interval same-different paradigm developed in a SDT framework (e.g. ^20,21^), in which listeners had to detect local and/or global interval changes between rhythmic melodic sequences that follow Western rules for pitch interval ratios. This allowed us to control for the respective influence of perceptual and decisional aspects in listeners’ responses, thus extending paradigms of previous studies in which these aspects remained confounded. In order to force participants listen to the whole stimuli, the modifications to-be-detected could occur either at the beginning or the end of the sequence, in an unpredictable manner. This design also allowed us to assess the extent to which global-to-local interferences depends on temporal aspects; changes occurring at different temporal positions may exhibit different results depending on hearing expertise, because musicians have developed stronger auditory memory capacities^22,23^. Lastly, inspired by the visual literature^2,24^, and in order to enrich the current description of auditory local–global processing, we tested how two stimuli characteristics not considered in previous studies—the contour of the pitch profile (e.g., rising, falling, or having a more complex shape) and the relative size of local vs. global pitch changes—modulate the observed effects.

## Materials and methods

### Participants

Fifteen expert musicians (nine female, six male; mean age: 20 ± 1.6 year) and fifteen non-musicians (eight female, seven male; mean age: 22 ± 2.4 year) were recruited for this study (no significant difference in age between groups, *p* > 0.05). Expert musicians were students from French musical institutions such as “Conservatoire National à Rayonnement Régional” (CRR) or “Conservatoire National Supérieur de Musique et de Danse de Paris” (CNSMDP), with at least 7 years of formal musical training and more than 2 h of everyday practice per day. Non-musicians were mainly composed of undergraduate students in psychology or neuroscience; responses to a musical experience questionnaire insured that they did not have any musical expertise or practice. All participants reported normal hearing, and had no history of audiological or neurological disorders. The study was approved according to Helsinki Declaration by the Ethics Committee of Institut Européen d’Administration des Affaires (INSEAD). All methods were carried out in accordance with their guidelines and regulations. Participants gave written informed consent and received financial compensation for their participation.

### Stimuli

**Figure 1:**
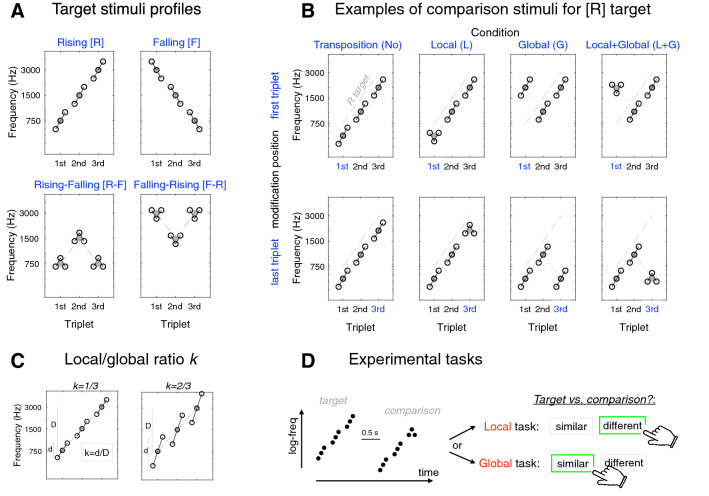
Stimuli and experimental task. (**A**) Four target stimuli profiles. In each sequence, the nine tones are represented by white circles, and the spectral centroid values of the three triplets by grey disks. Here, as in panel B, global profiles are shown by grey dotted lines, and local profiles by black full lines. (**B**) Examples of comparison stimuli associated to the rising [R] target (superimposed dotted line in each panel). The four columns show, from left to right, Condition No where there is only a pitch transposition, Condition L where there is a local modification, Condition G where there is a global modification and condition L + G where there are both local and global modifications; note that there is always a random pitch transposition. Modifications were made on the first (upper row) or on the last triplet (lower row). (**C**) Index introduced to compare the local and global scales: the local/global ratio k is the ratio of frequency ranges covered by a triplet (d) or the centroids of the three triplets (**D**); here, shown for the R target with values of 1/3 and 2/3. (**D**) Illustration of the experimental tasks. Participants heard one target followed by one comparison stimulus and had to determine whether the two were similar/different at a local level or at a global level (two separate tasks). They received trial-by-trial feedback. Here, correct responses are circled in green for the target and comparison stimuli shown as examples.

Stimuli construction was inspired from previous work, which used hierarchical melodies, i.e. their local and global pitch profiles differ in scale but not in shape (e.g. ^6,11,12^), with several notable differences. Here, each stimulus consisted in a sequence of 9 pure tones segmented into three-tone triplets. The local level was defined as the pitch structure within the triplets, and the global level was defined as the pitch structure formed by the mean pitch of the three triplets. There were two types of stimuli: target and comparison stimuli (see Fig. 1A,B). For target stimuli, the pitch profile, i.e. the interval directions formed by each triplet (“local” scale) and the profile formed by the values of the spectral centroids of the three triplets (“global” scale) were always similar. For comparison stimuli, this profile was kept identical for two triplets but could differ for one triplet (see details below). For both target and comparison stimuli, tones’ duration was 100 ms, inter-tone intervals within each triplet were 10 ms and inter-triplet intervals 120 ms, resulting in 1,200-ms sequences following a specific rhythm (sixteenth note triplets on every beat). Importantly, this introduced temporal segregation provides a natural separation between local and global scales, comparable to Navon’s original visual stimuli^1^. The frequency of the first tone was always chosen on a random uniform distribution, but sequences were in turn structured in order to respect specific musical intervals: there was always a difference of 4 or 8 semi-tones between two consecutive tones within a triplet (depending on the local/global change ratio considered; see “Procedure”), and there was always a one-octave difference between the centers of gravity (mean on a log-frequency scale) of the pitches between two consecutive triplets (see below). Tone levels were normalized in loudness across frequency using the ISO226 equal-loudness curve at 70 dB SPL^25^ to ensure they were similar in terms of perceptual emergence. All the stimuli were normalized in level and presented diotically at 70 dB SPL.

#### Target stimuli

We tested the full set of hierarchical patterns developed by Justus and List ^6^, extending the initial use of simple monotonic rising or falling pitch profiles (hereafter referred to as [R] and [F]) employed in previous studies^6,11,12^ to two novel non-monotonic rising-falling and falling-rising pitch profiles (hereafter referred to as [R-F] and [F-R]). Each triplet is indicated by C_j_ corresponding to the spectral centroid of the three tones within a triplet, *j* indicating its position within the sequence, from one to three. First, the value of the spectral centroid for the second triplet (C_2_) was randomly chosen following a random uniform distribution between 400 and 1,600 Hz (i.e. 2 octaves) for each trial. Then, values for the first (C_1_) and third (C_3_) triplets were ± 1 octave apart from the second one. To create the local pitch profile, the shape of the global profile was reproduced with a local/global ratio *k* (local/global pitch intervals on a log-frequency scale; see Fig. 1C) around each value of C_j_. We tested two different values of this local/global ratio: k = 1/3, as in previous studies^11,12^, and k = 2/3.

#### Comparison stimuli

Comparison stimuli were always different from target stimuli by at least a global pitch transposition (i.e. same on all tones) drawn from a uniform random distribution between ± 1 octave, and if more, by only one triplet (either the first or the last triplet to force listeners to attend to the whole sequence). We used four types of comparison stimuli: pitch transposition of the target stimulus only, local modification, global modification, or both local and global modifications (hereafter called Condition No, Condition L, Condition G and Condition L + G, respectively) (see illustration for the [R] profile, Fig. 1B). To ensure an independent manipulation of local and global modifications, the spectral centroid of the triplet where a local modification was applied to was preserved (Fig. 1B). When local and global modifications were applied simultaneously (Condition L + G), they occurred on the same triplet.

### Apparatus

The experimental session was run using Matlab on a Macintosh Mac Pro workstation with an RME Fireface 800 soundcard. Stimuli were created with an in-house Matlab program and were presented diotically through headphones (HD 280 PRO by Sennheiser electronic GmbH & Co. KG, Germany). The experimental setup was calibrated using a Brüel & Kjær 2238 Mediator sound-level meter, coupled with the mounting plate provided for circumaural headphones; a 1-kHz pure tone at a level of 70 dB SPL was used for the calibration. Each tone of a sequence was normalised in loudness accordance with EBU R 128 to be played at 70 phons. Each participant was tested in a double-walled IAC sound-insulated booth.

### Procedure

Since previous studies showed that the type of attention allowed by the task—attention-divided or attention-directed—had no effect on perceptual results and, neither for non-musicians nor for musicians ^12^, participants were tested in two distinct attention-directed tasks: a local and a global attention-directed task (see example in Fig. 1D) where they performed “similar-different” discrimination. In the local-directed task, they had to determine whether the local pitch profiles, i.e. the pitch contours of all three triplets of the comparison stimulus were similar to those of the target stimulus irrespective of its global pitch profile. In the global-directed task, they had to determine whether the global pitch profile, i.e. the pitch contour formed by the mean of the three triplets of the comparison stimulus was similar to that of the target stimulus irrespective of its local pitch profiles. In both tasks, listeners were asked to compare target and comparison stimuli in terms of pitch profile and had to ignore the overall pitch transposition between stimuli. This pitch roving procedure ensure that listeners focus on local/global pitch contours rather than the pitch of the sequences per se to make their judgment. Of note, our full factorial local (no/yes) × global (no/yes) design for the modifications ensured that listeners could only perform a given attention-directed task above chance by attending to the scale specified by the task. Target and comparison stimuli were separated by a 500-ms silent interval and presented once only. At the end of each trial, participants made their choice by pressing the appropriate “same”, or “different” buttons, and took as much time as they wished to respond. Participants received trial-by-trial feedback (correct/incorrect). The next interval followed the response after a fixed 500-ms delay.

Prior to the main experiment, participants familiarized with the task and the types of stimuli. We used auditory examples and visual analogies created for the specific purpose of the experiment (see Fig. S1) in order to avoid any explicit mention regarding how the local and global modifications were constructed. Next, participants underwent a training session with stimuli first presented two times slower to ensure that they understood and could easily perform the task in any condition, then at the same tempo as in the main experiment. While performing these training trials, participants could constantly refer to the visual illustrations of the different conditions proposed by the experimenter. The main session started once a participant could perform well above chance. This training session lasted approximately 15 to 30 min.

The proper local and global tasks were conducted in two separate sessions scheduled one day apart (order counterbalanced between participants). Taking into account the four variables–four profiles ([R], [F], [R–F] and [F–R]), four conditions (No, L, G, L + G), two positions for the pitch modification (first or last triplet), two local/global ratios (k = 1/3, k = 2/3)—there were 64 different configurations. In order to derive scores with enough precision, for each participant in each task, all 64 configurations were repeated 10 times, leading to a total of 640 trials. None of these trials were identical, since the pitch of the target was always drawn from a random uniform distribution. Each session was divided into 6 blocks, ensuring regular breaks throughout the experiment, and lasted approximately 1 h. Of note, the task and the way the stimuli were constructed were exactly identical for musicians and non-musicians; in other words, we did not adjust any stimulus parameter for each group.

### Analyses

This experiment is based on a 2 ×  [2 × 4 × 4 × 2 × 2] factorial design: one between-factor “Group” (musicians|non-musicians) and five within-factors “Task” (Local|Global)   ×  “Profile” (R|F|R–F|F–R) × “Condition” (No|L|G|L + G) × “Position” (First|Last)  ×  “Ratio” (k = 1/3|k = 2/3). We conducted two types of analyses. First, within the framework of SDT^18^, we computed confusion matrices to derive sensitivity (*d’*) and decision criterion (*c*) values for each participant, in each task, as a function of the modalities of the different factors (Table S1). Then, an *averaging* procedure yielded the overall sensitivity and decision criterion values (i.e. sensitivity and criterion values were first calculated separately for each combination of factor modality and observer, and then averaged (as recommended in^26^). For each task, participants’ responses were classified depending on the condition (see Stimuli):

- Local task: Hits = percentage of responses “similar” in Conditions No and G; False Alarms = percentage of responses “similar” in Conditions L and L + G.
- Global task: Hits = percentage of responses “similar” in Conditions No and L; False Alarms = percentage of responses “similar” in Conditions G and L + G.

Incidentally, in Condition No, one half of the “similar” responses (or respectively “different” responses) were associated to a modification of the first triplet, and the other half to the third triplet. According to these definitions, a negative (positive) decision criterion indicates that the participant favoured “similar” (“different”) responses.

In the present study, the local–global advantage and interference effects were defined using the performance scores obtained in Condition L and Condition G only, in order to rely on a definition of these indices comparable to that of previous studies^11,12,19^. We here define a global advantage as a better score (on average in Conditions L and G) in the global task compared to the local task, and a global-to-local interference when the performance in the local task is worst in Condition G (i.e. modification incongruent with the task) than in Condition L (i.e. modification congruent with the task); the statistical indices introduced later in the paper to assess advantage and interference effects in quantitative terms (i.e. in percentage values) correspond to these definitions. Thus, to assess advantage (local or global) and interference (global-to-local or local-to-global) effects, we simply conducted a mixed analysis of variance (mixed-ANOVA) on the performances obtained in Conditions L and G. Statistical significance thresholds were set at *p* < 0.05. Post-hoc t-tests were conducted when required, with Bonferroni corrections for repeating comparisons where appropriate.

## Results

We did not observe any learning effects over the time-course of the present tasks in both musicians and non-musicians, so all data were pooled together in the following analyses.

### SDT indices

**Figure 2:**
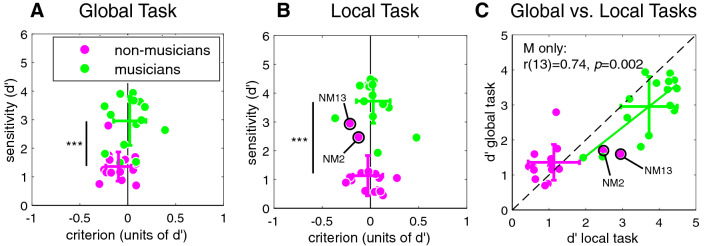
SDT indices. Individual sensitivity values plotted against individual criterion values for both groups (musicians, green; non-musicians, magenta) in the global (**A**) and the local (**B**) tasks. (**C**) Sensitivity correlates between local and global tasks for musicians (green regression line) but not for non-musicians. Two particular individuals from the non-musician group (NM2 and NM3) who exhibit similar results to musicians’ are highlighted.

We first asked whether musicians and non-musicians could perform the two tasks (local and global) above chance (sensitivity values *d’* > 0) and without any overall specific response strategy (criterion *c* ~ 0). The latter point is particularly important, because it guarantees that performance scores can be compared across conditions and across groups without any bias, as required for more in-depth analyses comparing scores across conditions and groups (next section). Overall sensitivity and criterion values in local and global tasks (Fig. 2A,B) confirmed that all participants could perform well above chance (t-tests comparing *d’* against zero, all Ps < 0.001) and had no specific response strategy (t-tests comparing *c* against zero, all Ps > 0.05, except in the global task for non-musicians where the criterion was slightly but significantly negative, *c* = − 0.10: t(14) = 2.86, *p* = 0.013).

We then compared sensitivity indices between groups and across tasks (Fig. 2C). First, we found that musicians outperformed non-musicians both in the global (t(28) = 6.20, *p* < 0.001) and the local (t(28) = 9.66, *p* < 0.001) tasks. Second, we found that musicians performed significantly better in the local task than in the global task (t(14) = 5.03, *p* < 0.001). There was an opposite trend for non-musicians to perform better in the global task than in the local task, but the difference was not significant (t(14) = 1.23, *p* = 0.24) (Fig. 2C: all individuals from the musicians group fell below the diagonal, while individuals from the non-musicians group fell both above and below the diagonal). Furthermore, in order to determine whether good (bad) performers in the global task were also good (bad) performers in the local task, we performed correlations between the sensitivity of the different individuals across the two tasks. We found a significant correlation for musicians (r(13) = 0.74, *p* = 0.002), but not for non-musicians (r(13) = 0.33, *p* = 0.23). Conversely, correlations between criterion values across the local and the global tasks were not significant for either group (Ps > 0.05). Beyond these group-level differences, it is also interesting to point out non-musicians NM2 and NM3 who exhibited similar sensitivities to musicians’ (these individuals are further highlighted throughout this paper).

### Performance scores across the four conditions

**Figure 3:**
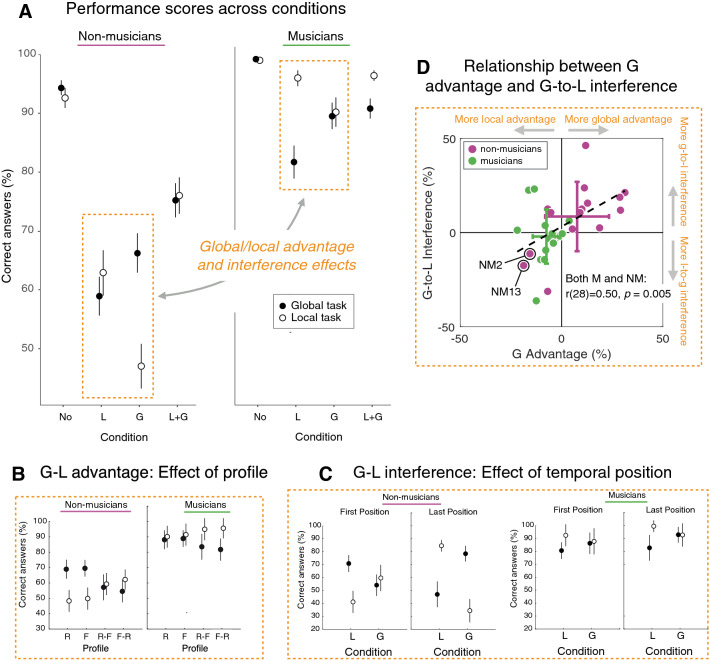
Performance scores. (**A**) Correct answers (%) for the two tasks and for the two groups as a function of the four conditions. Global–local advantage and global–local interference effects focus on L and G conditions highlighted by dashed rectangles (see main text for details). (**B**) Dependence of the global–local advantage on the type of target temporal profile. (**C**) Dependence of the global–local interferences on the temporal position of the modifications—1st or 3rd triplet. Error-bars show 95% confidence intervals. (**D**) Global advantage and global–local interference indices, and their relationship across individuals. Statistical indices corresponding to global advantage and global interference effects were computed for each participant (musicians, green; non-musicians, magenta). A correlation between these two indices, mainly driven by the individuals from the non-musicians group (see the text), was found. Error bars show mean and SD of each group along the two dimensions. Non-musicians NM2 and NM13 are highlighted.

In order to assess how local or global modifications specifically impacted listeners’ performance depending on their group and the task, we first consider in details participants’ performance scores (percentage of correct answers) across Condition L and Condition G (see dashed rectangles in Fig. 3A). Performances obtained in these two conditions were analyzed with a mixed-ANOVA (see "Materials and methods"). All significant effects and interactions are presented below. Incidentally, the Ratio factor neither had significant main effect nor interacted with other factors. In line with the results from SDT indices, results showed that musicians outperformed non-musicians when considering conditions L and G (Fig. 3A), as supported by a significant difference between the two groups (F(1, 28) = 70.9, *p* < 0.001). Consistent with the definitions of previous studies and as detailed above, we assessed advantage and interference effects by focusing on performances restricted to Conditions L and G.

#### Global advantage for non-musicians with [R] and [F] profiles and local advantage for musicians with [R–F] and [F–R] profiles

Non-musicians exhibited better scores in the global task than in the local task, and the opposite pattern was observed for musicians (Fig. 3A), as supported by a Group x Task interaction (F(1, 28) = 11.6, *p* < 0.01). Furthermore, this pattern was modulated by the type of Profile (see Fig. 3B), as supported by a significant Group x Task x Profile interaction (F(3, 84) = 4.75, *p* < 0.01). Non-musicians had significantly higher scores in the global task compared to the local task for [R] and [F] profiles (Ps < 0.05), but similar scores in both tasks with [R–F] and [F–R] profiles (Ps > 0.05). In contrast, musicians had similar scores in the local task and global tasks with [R] and [F] profiles (Ps > 0.05) and higher scores in the local task compared to the global task with [R–F] and [F–R] profiles, although it did not reach significance (Ps > 0.05). Overall, these results (i) show a global advantage for non-musicians to process monotonic ([R] and [F]) profiles and (ii) suggest a trend toward a local advantage for musicians to process non-monotonic ([R–F] and [F–R]) profiles.

#### Global-to-local interferences modulated by the temporal position of the modification

Statistical analysis revealed a significant Group × Task × Condition × Position interaction (F(1, 28) = 46.48, *p* < 0.01; Fig. 3C). This means that the interaction between Condition (L or G) and Task (local or global) was not the same for non-musicians and musicians, and that it was in addition modulated by the temporal position of the modifications (first or last). For musicians, the pattern of scores related to the global-to-local interference effect was unaffected by the temporal position of the modification (Fig. 3C, right panel). In contrast, the pattern of scores for non-musicians was reversed depending on whether modifications occurred on the 1st or the 3rd triplet (Fig. 3C, left panel). Detailed analyses of SDT indices suggest that this pattern reversal can be explained by the specific response strategy adopted to cope with the higher difficulty experienced when modifications were on the first vs. last triplet (see Supplementary Information). Overall, these results show that there was no trend for global-to-local or local-to-global interference effects in musicians, and that the apparent modulation of the global-to-local interference in non-musicians regarding the temporal position of the modifications could be explained by their response strategy.

#### Relationship between global advantage and global-to-local interference

To assess the global advantage and global-to-local interference effects as well as their relationship more quantitatively, we computed two indices that directly reflect these effects. With **S**_tc_ the mean score (percent of correct responses) of one participant in task *t* and condition *c*, with *l* and *g* referring to the local and global tasks/conditions, the index for global advantage was computed as the difference between global and local scores [G advantage = ½ × (S_gl_ + S_gg_) − ½ × (S_ll_ + S_lg_)], and the index for global–local interference was computed as the difference between global-to-local interference and local-to-global interference effects [G-to-L interference = (S_ll_ – S_lg_) − (S_gg_ − S_gl_)]. The values of these two indices obtained for the different participants, which can be read as percentage score units, are plotted against each other in Fig. 3D. Musicians and non-musicians cluster in two separate groups with little overlap, highlighting the observed global advantage and global-to-local interference effects previously presented. Importantly, we found an overall significant positive correlation between global advantage and global interference indices (r(28) = 0.50, *p* = 0.005), which was driven by data from non-musicians (r(14) = 0.65, *p* = 0.008), not from musicians’ (r(14) = − 0.03, *p* = 0.91). This suggests that, at least for non-musicians, there would be a relationship between these two perceptual phenomena.

#### Further group differences in local–global processing in light of condition no and condition L + G

The two remaining experimental conditions (Condition No and Condition L + G), which were introduced to force listeners to direct their attention locally or globally in the two experimental sessions (see “Procedure”), are also informative to interpret their strategies. In Condition No, performance was close to ceiling for musicians and slightly lower for non-musicians, showing that all listeners were able to correctly perform a comparison between target and comparison stimuli in the most simple condition (pitch transposition between both stimuli). In Condition L + G (simultaneous local and global modifications of the comparison stimulus), musicians’ scores in the local task were similar to those obtained in Condition L and in the global task they were similar to those obtained in Condition G. In other words, when there were local and global modifications at the same time, musicians performed similarly as when there was only one modification at a time congruent with the task (i.e. local modification in the local task or global modification in the global task). In contrast, non-musicians’ scores in both the local and global tasks were unexpectedly higher to those obtained in Condition L and Condition G. In other words, when there were local and global modifications at the same time, non-musicians performed better than when there was only one modification at a time. Overall, these results suggest that while musicians are always able to identify the type of modification (local or global), non-musicians are able to detect that there is a modification but show some degree of confusion when it comes to identify whether it is local or global (which explains their lower performances in Conditions L and G compared to Condition L + G).

## Discussion

In the present study, we re-addressed the question of local–global reorganization in naive listeners and expert musicians using a novel same-different paradigm, in which participants had to detect local and/or global modifications between rhythmic melodic sequences following musical intervals with different profiles. The main goal of this study was to assess potential interferences between the scale at which listener’s attention is directed (local or global task) and the scale(s) at which the modification(s) occur (local and/or global conditions).

### The global precedence effect: an initial property of the local–global processing organization that is reshaped with musical training

#### Effects from previous studies (partially) replicated using another experimental design

In line with a myriad of previous psychophysical works (see^14^), our study showed that musicians outperformed non-musicians in both local and global tasks (Figs. 2 and 3). This corroborates observations made in the music cognition literature concerning local/global processing, namely that musicians show an increased ability to process both intervals and contours^15^, and that extensive music training develops both global and local music processing abilities^27^. However, our main interest here was to characterize how the pattern of performance scores differed across conditions between the two groups. First, in line with^10,12^, our data provide new evidence that non-musicians exhibit global advantage and global-to-local interference effects (see above for a definition of these effects) to detect local/global modifications in melodic sequences made of monotonic pitch variations (rising or falling). In contrast, we found no evidence for global advantage or global-to-local interference effects in musicians for this type of monotonic stimuli. This latter result differs from^12^, where a reduction but not a full suppression of the global-to-local interference effect was observed. However, it is important to note that both the paradigm and the stimuli design differed between our studies. First, we employed a same-different task in which listeners had to *compare* two sequences (and not to identify the pitch direction in one isolated sequence). Second, we employed rhythmic tone sequences structured with musical intervals (and not melodic sequences with regularly spaced notes following non-musical intervals). As pointed out in the introduction, we designed the stimuli for the specific purpose of maximally exposing the local–global processing reorganization induced by musical training. Thus, our results already suggest at this stage that the local–global processing organization depends on stimuli and/or task characteristics. Importantly, our experimental design involved many other factors that were not considered in previous studies^10–12^, which now allow us to better ascertain the conditions that govern global advantage and global-to-local interference effects. The respective influence of these factors is discussed below.

#### Pitch profile complexity reveals a distinct musicians / non-musicians organization

Previous studies investigating the local–global organization only used hierarchical pitch sequences varying monotonically, namely rising or falling^11,12^. Here, using a richer set of pitch profiles, we found that non-musicians exhibit a global advantage solely with monotonic [R] or [F] profiles (i.e. monototic), and a trend toward local advantage is observed for musicians with [R–F] and [F–R] profiles (i.e. non-monotonic). In other words, this suggests that the global advantage actually depends on the complexity (i.e. monotonic vs. non-monotonic) of the hierarchical profiles, and that this interacts with musical expertise. As such, these results allow us to further specify the generally adopted view that ‘global information is treated more efficiently and strongly impairs local processing’ only applies to the case of simple monotonic patterns processed by naive listeners. These results could be related to the different degrees of prediction associated to these patterns. While the global profile of monotonic [R] and [F] patterns might be easily accessed by non-musicians based on e.g. implicit heuristics and Gestalt rules, building a representation of the global profile of non-monotonic [R–F] and [F–R] patterns might be much less trivial, and as such favour the processing to the local information. The musician-non-musician different weighting of global and local processes would thus be maximally exposed by these different profiles complexity.

#### The size of local pitch intervals does not impact local–global processing

Previous studies in vision had shown that although the absolute size of local and global elements had no measurable impact on local–global processing characteristics^1^, their relative size appeared to be a major determinant of global advantage, with bigger global advantage effects observed for larger global/local ratio^2^. Consistent with these results, one study in the auditory domain^7^ reported reduced global advantage and reduced global-to-local interference effects when the local/global ratio of the stimuli was increased. In contrast, our data did not show any measurable influence of the local/global ratio on performances, in particular it did not impact global advantage and global-to-local interference effects. This apparent discrepancy might stem from how the local/global ratio was manipulated. In^7^, the local intervals remained constant while the size of the global pitch change was manipulated, whereas in the present study, we manipulated the size of the local intervals, not the global size that was always equal to ± 1 octave. This would be consistent with previous works in vision suggesting that the global precedence effect can be modulated by the difficulty of the global perception task, which actually differs when the global changes differ in size (see^7^).

#### Non-musicians’ difficulty to parse local and global modifications

While out of the initial focus of the present study, the results obtained in Condition L + G (see Fig. 3A), i.e. when local and global modifications were applied together, allow us to enrich our interpretations. This analysis was not possible in previous studies that only modified either the local or the global levels of the stimuli, not both at the same time. Our results suggest that while musicians are able to *identify* the type of modification, i.e. local or global (performances in Condition L and in Condition G predict performances in Condition L + G), non-musicians can *detect* that there is a modification but show a certain degree of difficulty to identify whether it was local or global (performances in Condition L and in Condition G are lower than performances in Condition L + G). Indeed, this pattern of scores is consistent with non-musicians responding more often “different” (to increase their scores) when they are able to detect that there is modification, even though they cannot identify it. These results interestingly suggest that musicians are able to filter out information that occur at the other level from which their attention is directed to (e.g. local modification in the global task). Put differently, local and global processing would operate independently in musicians, with little or no interference. In contrast, non-musicians would rather deploy a strategy that integrate both local and global modifications together, which would allow them to reach a better detection at the cost of not correctly identify whether it is local or global, leading to the lower performances observed in Conditions L and G compared to Condition L + G.

### Candidate mechanisms governing local–global processing reorganization

Which specific mechanism(s) do individuals develop through extensive musical practice make them performing our auditory task so differently? Our task involves a wide range of low-level and high-level capacities: listeners have (i) to encode the pitch profile of the stimuli, (ii) to store in memory the local or global patterns of the target stimulus (depending on the task at hand), (iii) to selectively attend the same level(s) of information in the comparison stimulus (appropriate direction of auditory attention), and (iv) compare these two pieces of information to decide whether they are similar/different (prediction and selective attention^28^). Therefore, addressing this question would require testing the same participants in many other more specific tasks. Nevertheless, several higher-level mechanisms appear to more likely contribute to the observed differences.

First of all, it is important to emphasize that, given the duration of the stimuli in the present study, the local/global interference effects highlighted here should be best understood in the context of *relative* temporal organizational principles of the auditory system irrespectively of the absolute temporal characteristics or tempi of the melodic sequences (see^29^). It has to be distinguished from studies concerned with the properties of *absolute* local–global temporal windows of the auditory system, which can nevertheless exhibit similar types of interactions^30,31^. Yet, the temporal distance between tones and in particular between the defined triplets is an important timing aspect of the stimuli that should be considered in future works, as it could likely be a limiting factor in the formation and processing of local “objects”, especially for non-musicians listeners. One interesting way of further scrutinizing the time-course of local–global temporal processes recruited in the present context could be to consider sequences of greater temporal complexity, e.g. by inserting silent intervals of different duration within triplets and between triplets, and manipulate the ratio of these two intervals (see^21,31^ for similar protocols).

The contribution of bottom-up aspects of pitch encoding to account for the differences between musicians and non-musicians should be minimal. Indeed, the smallest pitch changes to be detected correspond to a 2-octave shift in the global task, and equal or larger than 400 cents in the local task, which is largely higher than the just-noticeable-differences (JNDs) typically measured with isolated pure tones in naive listeners^32^. Rather, the present results can best be understood in the context of “informational masking”, in line with studies reporting JNDs of several thousands hertz when measured using random tone sequences varying in large frequency ranges^33^. In other words, this tells us how listeners’ processing are impacted when local/global pitch changes to be detected are embedded within longer sequences where other pitch changes can co-occur on the unattended level. Thus, the main musicians/non-musicians processing difference observed does not correspond to a mere difference in pitch sensitivity, but rather reflects a difference in their ability to independently integrate and process local pitch changes (i.e. within triplets) in the presence of interfering changes on the overall pitch contour (i.e. across triplets). As such, it should be interpreted as a reorganization of priority rules underlying local/global temporal processing.

Furthermore, we used melodic sequences with specific musical-interval structures, and in particular with ± 1 octave between triplets (see “Procedure”). Thus, our data need to be considered in relation with the phenomenon of “octave equivalence”, which corresponds to the acquired perceptual equivalence of intervals on a logarithmic scale with musical training (e.g.^34^); an effect strong enough that it does not require musical stimuli and can be observed with pure tones^35^. Thus, one might hypothesize that the suppression of the global advantage effect observed for musicians has to do with the fact that the pitch difference between consecutive triplets and any global modifications was always ± 1 octave, which corresponds to the most perceptually similar interval for expert listeners. Further studies involving different types of pitch intervals (e.g. non-musical, musical matching/not matching any octave relationship) need to be conducted to address the extent to which the octave equivalence effect contributes to our results. Comparing the response patterns of western and non-western musicians in two versions of the present protocol, with local and global changes complying to the western musical scale or to random non-integer pitch ratios would be of particular interest, since the phenomenon of octave equivalence is found to be less pronounced in non-western listeners^34^. Besides, we here employed a random frequency transposition between target and comparison sequences to avoid that listeners would compare sequences directly using pitch, but instead focus on the pitch structures of the two sequences. Further experiments could deploy this paradigm using FM-tones, or narrow-band noises that do not elicit a pitch sensation, to investigate the extent to which pitch per se contributes to the observed local/global organization and differences between musicians and non-musicians^21^. It is also important to note that individuals’ characteristics others than musical experience could influence the present results. For example, it could be that people speaking or having experience with tonal languages (e.g. Mandarin) might show enhanced global processing and less local-to-global interference, as tonal languages have a global pattern riding on the top of local units (phonemes) to convey linguistic information. Whether experience with tonal languages transfers to general auditory temporal processing organization, similarly as musical learning favours the development and prioritization of local processing, is a very interesting hypothesis that future studies could assess with the present protocol.

Lastly, the observed musicians vs. non-musicians differences could also be related to memory and attentional mechanisms. There is long-standing evidence for increased short-term auditory memory in musicians regarding pitch changes^36^, and more generally, musical expertise is associated with enhanced pitch and time processing for both music and speech^14,37^. Yet, if memory is at the basis of these differences, it is not clear whether these differences are related to the memory capacity itself or the way information is encoded. It might be possible that naive listeners best encode Gestalt characteristics compared to more detailed information^22^, whereas expert musicians are able to form and encode an abstract representation of both scales^15^.

Furthermore, musical training is known to enhance selective attention capacities and reduce prefrontal response variability^38,39^, which could also explain why global/local interferences are not observed in musicians. It would also be interesting to examine, as in vision, the extent to which this global/local privilege is constrained by the respective saliency of the different levels of information^4,40^, and whether this stimulus saliency is the factor that is modulated by the degree of musical expertise. Interestingly, in the visual domain, studies have shown a comparable reorganization of local–global processing between art vs. non-art students^41^ as the one evidenced in the present study, and found that observers following an extensive training to attend to the local features in a global/local visual task can result, after thousands of trials, in a local–global processing reorganization, where global advantage is suppressed and the global-to-local interference significantly reduced^42^. They hypothesized that training would make learn to inhibit their initially favoured processing of the global form (“global inhibition hypothesis”^42^). Whether a comparable process is at play following long-term musical training is an empirical question that should be addressed. Interestingly, our data reveal a relationship between the global advantage and global-to-local interference effects, suggesting that these phenomena might actually be mediated by the same underlying mechanisms, at least for non-musicians (see Fig. 3D). Future studies will have to determine whether the effects modulated by musical expertise revealed by the present study originate from the acquisition of enhanced short-term memory or enhanced ability to deploy selective attention, i.e. the ability to focus on particular aspects of a scene, which are in the present case tightly linked. Incidentally, the fact that some non-musicians individuals had similar results to experts (see Fig. 3D) might suggest that local–global re-organization is not mediated by mechanisms solely improved through *extensive* music learning^43^, thus pointing out toward more general cognitive developments.

## Conclusion and perspectives

All together, our results provide new evidence that the global precedence effect described so far for the processing of local/global melodic sequences does not constitute a “rigid” property of human perceptual organization, as it strongly varies for individuals with different degrees of musical expertise and depends on the complexity of the pitch profile considered. We provide further evidence that the initial “global bias” observed in most listeners when processing musical stimuli can be reversed or cancelled with training, leading to the acquisition of enhanced local processing or “analytical” listening abilities in musicians^44^. Yet, while proper musical training might strongly develop such analytical abilities, it might not constitute a necessary condition; futures studies will have to pinpoint which specific mechanisms underlie this re-organization. Interestingly, this trend toward a local-advantage for musicians is comparable to results obtained with ASDs who also exhibit enhanced analytic auditory capacities^45^; a link between the underlying local–global processing reorganization of musicians and ASDs has even been recently advanced^19^.

Our study is a first step toward a better understanding of the local–global processing perceptual reorganization in musicians that opens many perspectives for future works. The methodological development of this study responds to the need of the community for new paradigms “to overcome restrictions of classical global-to-local paradigms”^19^. It could thus be valuable for future electrophysiological or neuroimaging studies to re-examine the hemispheric specialization and, more generally, the neural local–global processing organization in different population^8,9^, with a control on both sensitivity and response strategy. Lastly, it would be interesting to determine whether the present parametric paradigm could be turned into an auditory tool for rehabilitation purposes, e.g. to restore the local–global processing when it is presumably impaired, such as for speech processing in dyslexics children. Because of the close relationship between melodic information and prosody processing in speech^37,46,47^, it might turn out to be more efficient than current visual paradigms ^48^.

## Supplementary information

Supplementary Information.
