# Supplementary material for: Auditory local–global temporal processing: evidence for perceptual reorganization with musical expertise
Source: Sci Rep. 2020 Oct 2;10:16390. doi: 10.1038/s41598-020-72423-7 (PMC7532159; doi:10.1038/s41598-020-72423-7)
Supplement: Supplementary file 1 — Supplementary Information. [file 41598_2020_72423_MOESM1_ESM.docx]

**Supplementary Material**

**Auditory local-global temporal processing: Evidence for perceptual reorganization with musical expertise**

Patrick Susini, Sarah Jibodh Jiaouan^,^ Elena Brunet, Olivier Houix, Emmanuel Ponsot


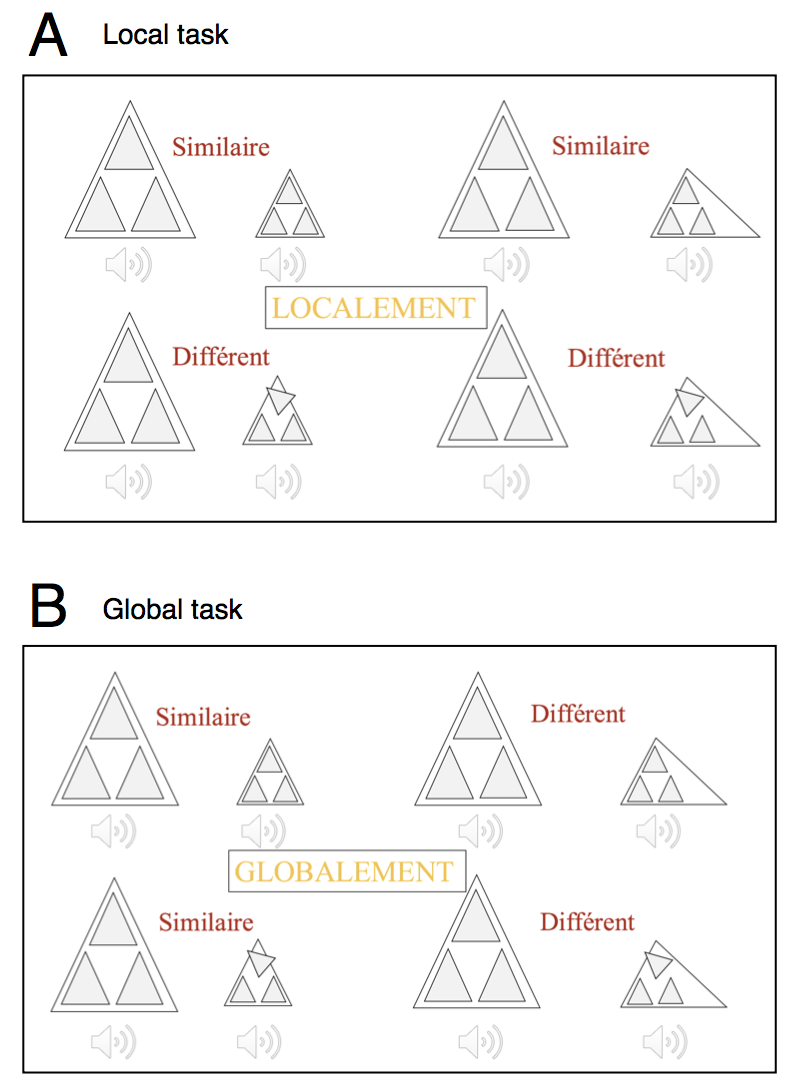


**Figure S1**. **Visual drawing analogies presented with experimental instructions.** During the instruction phase and the practice session, participants were presented with visual analogies (text in French) that were specifically developed for the purpose of the task, to help them *all* – i.e. both musicians and non-musicians, who can be unfamiliar with the concepts of local and global information within an auditory sequence – understand the local (panel A) and global (panel B) tasks. For these illustrations, the global information was symbolized by a large triangle, and the local information by three smaller triangles inserted in the large triangle (corresponding to the three triplets). The size of triangles was varied in all conditions, to reflect that there was at least a pitch transposition; a change of one small triangle symbolized a local modification, a change of the large triangle symbolized a global modification; a change of both the small and the large triangles symbolized modifications at both local and global levels. The expected answers for these different conditions are mentioned according to each task (text in French).

**Supplementary Information - Disambiguating the effect of temporal position on performance scores from detailed analyses of SDT indices**

In order to further interpret the interaction found between the temporal position of the modifications and the global-to-local interference pattern (see Fig. 2C), we conducted complementary analyses aiming to disembiguate participants’ sensitivity and response strategies deployed according to this factor. We reasoned that if listeners adopt a particular response strategy depending on the position of the modification, e.g. by answering more “similar” or “different” for a given position, it might complicate the interpretation of their pattern of scores, and as such would not transparently reveal any *true* change in local-global processing organization. According to how confusion matrices were computed (see Analyses), a shift of the criterion from zero (i.e. neutral strategy) would indeed correspond to an increase (decrease) of Hits and a decrease (increase) of Correct Rejections, which is the type of pattern reversal that we observed between the two temporal position of the modifications for non-musicians (see Fig. 2C).

To do so, we relied on detailed SDT indices computed according to the main contributing factors (Table S1), and analysed in particular the sensitivity and criterion values according to Position factor, for both tasks.

|  |  | Sensitivity (*d’*) | |  | Criterion (c) | |
| --- | --- | --- | --- | --- | --- | --- |
|  |  | Non-M | M |  | Non-M | M |
|  |  |  |  |  |  |  |
| Task | Local | 1.13 (0.70) | 3.72 (0.77) |  | -0.03 (0.15) | 0.03 (0.18) |
|  | Global | 1.36 (0.51) | 2.96 (0.86) |  | -0.10 (0.13) | 0.02 (0.17) |
|  |  |  |  |  |  |  |
| Position | 1^st^ triplet | 1.03 (0.49) | 2.99 (0.70) |  | -0.41 (0.25) | -0.04 (0.11) |
|  | 3^rd^ triplet | 1.72 (0.56) | 3.40 (0.55) |  | 0.35 (0.13) | 0.06 (0.13) |
| Task x Position | Local, 1^st^ triplet  Local, 3^rd^ triplet  Global, 1^st^ triplet  Global, 3^rd^ triplet | 0.83 (0.72)  1.73 (0.78)  1.22 (0.50)  1.71 (0.55) | 3.27 (0.79)  3.69 (0.33)  2.71 (0.76)  3.11 (0.82) |  | -0.41 (0.33)  0.45 (0.19)  -0.40 (0.27)  0.25 (0.17) | -0.02 (0.21)  0.08 (0.15)  -0.06 (0.19)  0.05 (0.19) |
| Profile | R | 1.26 (0.51) | 3.36 (0.79) |  | 0.00 (0.16) | 0.11 (0.17) |
|  | F | 1.33 (0.43) | 3.42 (0.86) |  | 0.00 (0.15) | 0.11 (0.14) |
|  | R-F | 1.20 (0.59) | 3.34 (0.81) |  | -0.14 (0.16) | -0.03 (0.17) |
|  | F-R | 1.20 (0.54) | 3.24 (0.71) |  | -0.11 (0.19) | -0.08 (0.20) |

**Table S1**. **Sensitivity (*d’*) and criterion (c) indices.** These indices are computed as a function of the task, the position and the target profile, both for non-musicians (Non-M) and musicians (M). Values indicate Mean (SD).

Regarding sensitivity values, we found lower performance when the modifications occurred on the first vs. last triplet for both groups and in both tasks (t-tests, all Ps<0.05). Regarding criterion values, we found that non-musicians applied different criteria between the two positions both in the local task (t(14)=7.42, *p*<0.001) and in the global task (t(14)=7.01, *p*<0.001), whereas musicians did not, neither in the local task (*p*>0.05), nor in the global task (*p*>0.05).

First, these results imply that for musicians, who did not adopt any specific response strategy, their performance scores can be readily interpreted and we can reasonably conclude that the position of the modification did not have any effect on their global-local interference pattern.

In contrast, these results indicate that, in both tasks, non-musicians used significantly different response strategies depending on whether the modifications occurred on the first or the last triplet. Importantly, this first implies that non-musicians were able to detect when (or where) the modifications occurred, on the first or on the last triplet: indeed, if they were not able to identify where the changes occurred prior to identifying which change it was, the response criterion would necessarily be the same for these two positions. In order to determine whether this shift in decision criterion was associated with the evidenced higher difficulty (i.e. differences in sensitivity) observed for correctly detecting the modifications on the first than on last triplet, we performed Pearsons’ correlations between changes in sensitivity and changes in decision criteria between these two positions. This correlation was highly significant in non-musicians in the local task (r(14)=0.76, *p*<0.001) and only marginally in the global task (r(14)=0.53, *p*=0.04). In contrast, none of these correlations were significant for musicians (local task: *p*>0.05; global task: *p*>0.05). These analyses demonstrate a strong relationship between the differences in sensitivity observed between the two positions and the change in decision criteria in non-musicians: the larger the difficulty was for detecting a change in the 1st triplet compared to the 3rd triplet, the less neutral was the response strategy adopted by non-musicians’ listeners. In other words, the specific strategy adopted by non-musicians to cope with this disparity in difficulty across the two positions was to give more “similar” answers when they could identify that the change occurred on the 1st triplet, and inversely, to give more “different” answers when they could identify that the change occurred on the 3rd triplet. The fact that this phenomenon is only observed in the local task, not the global task, could be attributed to the global-to-local interference effect.

Going back to our initial problem concerning the interpretation of the reversal of the pattern of scores observed in non-musicians, results from these SDT analyses imply that their performance scores do not return a transparent information of the global-to-local interference pattern, it is distorted by the non-neutral response strategy. This non-neutral response strategy adopted by non-musicians prevents any direct interpretation of the temporal position effects on the reverse of their patterns of scores, and thus do not allow us to determine whether (and, if so, to which extent) the local-global processing truly depends on the position of the modifications. Hence, at present, we can only provide a *qualitative* interpretation of the effect of the temporal position observed on the global-to-local interference effect for non-musicians: this reorganization appears to be (at least partly) driven by an increased tendency of non-musicians to report in the local task that the two stimuli were similar (different) when they could identify that the change was on the first (last) triplet.

Motivated by the insights offered by a detailed inspection of SDT indices across positions, and in order to reach a deeper understanding of the underlying factors driving the observed performance differences between musicians and non-musicians, we performed full correlational analyses between sensitivity (*d’*) and criterion values (c) computed across tasks and positions of the modifications separately (1^st^ or 3^rd^) (Fig. S2). These analyses exhibited very different patterns between non-musicians and musicians, which are discussed below.

**
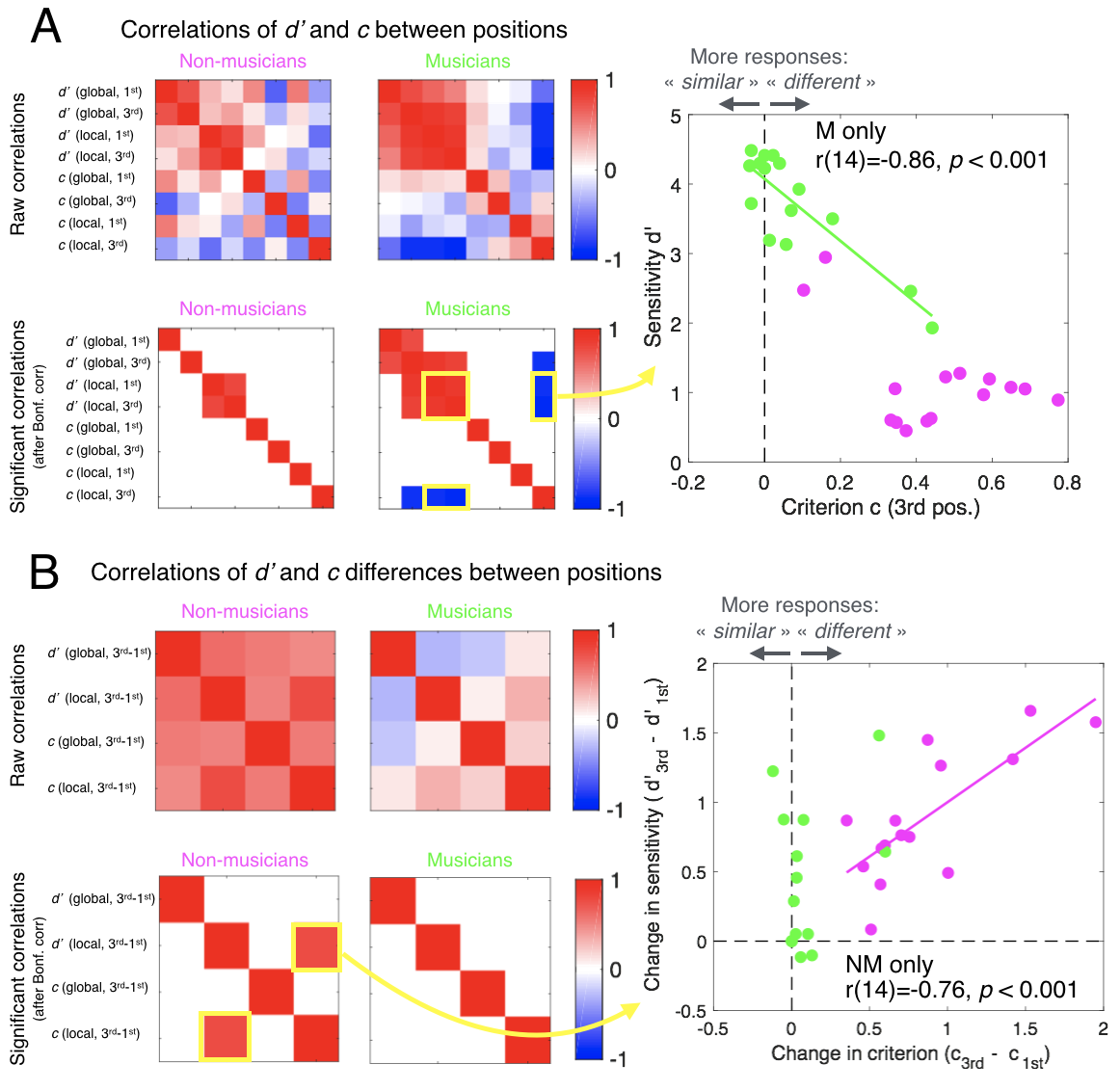
**

**Figure S2**. **Correlational analyses from SDT indices across conditions.** A) Pearsons’ correlations across sensitivity and criterion values derived in the local and global tasks for the two positions (1^st^, 3^rd^) (left: non-musicians, right: musicians; upper panels: raw Pearson correlation values; lower panels: Pearson correlation values remaining at *p* < 0.05 after Bonferroni correction for N=36, i.e. the total number of tested correlations). In the local task and for musicians only, the sensitivity is correlated with the criterion applied when the modification was on the 3^rd^ triplet (i.e. zones highlighted by yellow rectangles in panel A) are correlated for the musician group only. B) Pearsons’ correlations of the *differences* in sensitivity and *differences* in criterion values between the different positions (3^rd^-1^st^) for local and global tasks (same legend as in A). In the local task and for non-musicians only, change in sensitivity is correlated with change in criterion.

First, we considered Pearsons’ correlations across all sensitivity and criterion values in the local and global tasks for the two positions (Fig. S2A, left panels). These correlational matrices did not show any significant relationship between sensitivity and criterion indices for non-musicians, whereas for musicians performing the local task, there was a negative relationship between the overall sensitivity and the response criterion when the change occurred on the 3^rd^ triplet (bottom left corner of the matrix highlighted by yellow rectangles). This correlation was highly significant for musicians (r(14)=-0.86, *p*<0.001), but did not reach significance for non-musicians (r(14)=-0.49, *p*=0.06). To visualize this relationship, we plotted the overall sensitivity of all participants in the local task as a function of their specific response criterion when the modification occurred on the 3^rd^ triplet (Fig. S2A, right panel). This plot shows that there is little overlap between the two groups, and highlights the fact that the correlation that only holds true across musicians. This effect is particularly intriguing as it implies that musicians’ performance in the local task can be predicted by the specific response criterion deployed when they identified that the change occurred on the last triplet. More specifically, it suggests that this tendency to respond “different” in this particular condition is associated with a lower performance.

Second, we considered Pearsons’ correlations computed from *differences* in sensitivity and criterion values between the two positions (Fig. S2B, left panels), i.e. in a similar way as what we did above when relating sensitivity differences and criterion differences across positions. In contrast, these analyses did not exhibit any significant relationship between sensitivity and criterion indices for musicians whereas, for non-musicians, they showed that the only significant relationship was the one discussed above between their changes in performance and their changes in decision criteria between the two positions in the local task (Fig S2B, right panel).

Taken together, these correlational analyses between sensitivity (or changes in sensitivity) and decision criteria (or changes in decision criteria) evidence a clear dissociation between musicians and non-musicians, specifically in the local task. When a change occurred on the last triplet, musicians were on average able to make their decision without applying any specific decision strategy while non-musicians favoured the response “different” (Fig. S2A, right panel). Yet, our data suggest that the same effect was present in some musicians’ listeners, whose sensitivity appeared to be constrained by the same response strategy (negative relationship between sensitivity and response strategy). This specific relationship was not observable when a change occurred on the first triplet. Conversely, our results suggest that another phenomenon based on the *difference* of processing between the first and last triplet is at play for non-musicians. This musician vs. non-musician dissociation is observed only in the local attention-directed task, which could be explained by the fact that this level of processing is most impacted by musical training. Future studies should investigate how the sensitivity and criterion measures derived from the present task relate to more general memory and attentional capacities.
